# BRAF-Mutated Non-Small Cell Lung Cancer: Current Treatment Status and Future Perspective

**DOI:** 10.3389/fonc.2022.863043

**Published:** 2022-03-31

**Authors:** Ningning Yan, Sanxing Guo, Huixian Zhang, Ziheng Zhang, Shujing Shen, Xingya Li

**Affiliations:** Department of Medical Oncology, First Affiliated Hospital of Zhengzhou University, Zhengzhou, China

**Keywords:** BRAF, NSCLC, targeted therapy, immune checkpoint inhibitors, tyrosine kinase inhibitors

## Abstract

V-Raf murine sarcoma viral oncogene homolog B (*BRAF*) kinase, which was encoded by *BRAF* gene, plays critical roles in cell signaling, growth, and survival. Mutations in *BRAF* gene will lead to cancer development and progression. In non-small cell lung cancer (NSCLC), *BRAF* mutations commonly occur in never-smokers, women, and aggressive histological types and accounts for 1%–2% of adenocarcinoma. Traditional chemotherapy presents limited efficacy in *BRAF*-mutated NSCLC patients. However, the advent of targeted therapy and immune checkpoint inhibitors (ICIs) have greatly altered the treatment pattern of NSCLC. However, ICI monotherapy presents limited activity in *BRAF*-mutated patients. Hence, the current standard treatment of choice for advanced NSCLC with *BRAF* mutations are *BRAF*-targeted therapy. However, intrinsic or extrinsic mechanisms of resistance to *BRAF*-directed tyrosine kinase inhibitors (TKIs) can emerge in patients. Hence, there are still some problems facing us regarding *BRAF*-mutated NSCLC. In this review, we summarized the *BRAF* mutation types, the diagnostic challenges that *BRAF* mutations present, the strategies to treatment for *BRAF*-mutated NSCLC, and resistance mechanisms of *BRAF*-targeted therapy.

## Introduction

Lung cancer is the most commonly diagnosed cancer and the leading cause of cancer mortality in China (1). Lung cancer could be divided into non-small cell lung cancer (NSCLC) and small cell lung cancer (SCLC); of these, approximately 60% of NSCLC were adenocarcinoma (2). Generally, about 80% of lung adenocarcinoma harbors driver mutations in east Asians (3). Over the past decades, targeted therapies have dramatically revolutionized the treatment pattern of NSCLC and greatly improved the prognosis of NSCLC patients.

V-Raf murine sarcoma viral oncogene homolog B (*BRAF*) gene encodes *BRAF* kinase, a member of mammalian cytosolic serine/threonine kinases, which plays important roles in cell signaling, growth, and survival (4–6). *BRAF* mutations are rare mutations in NSCLC, which account for 2% of lung adenocarcinoma, and more frequently occur in never-smokers, women, and aggressive histological types (micropapillary) (7). Additionally, *BRAF* V600E mutations are mostly mutually exclusive with most druggable abnormalities present in this tumor (8, 9). It should be noted that certain *BRAF* mutations can coexist with *KRAS* mutations (9). However, routine platinum-based chemotherapy presents lower efficacy and is associated with poorer survival (10). Currently, the advent of BRAF inhibitors (BRAFi) and immune checkpoint inhibitors (ICIs) has transformed the landscape of *BRAF*-mutated NSCLC. In the present review, we will discuss *BRAF* biology within the context of oncogenesis. In addition, we will describe the evolving science of molecularly targeted therapies and ICIs for *BRAF*-dependent cancers.

## BRAF Mutations in Cancer

**Figure 1:**
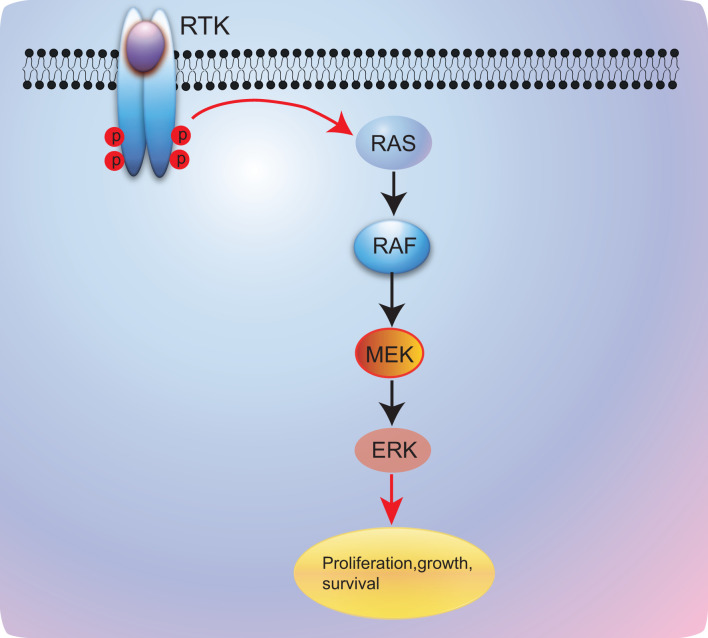
RAS/RAF/MEK/ERK signaling pathway. RTK, receptor tyrosine kinase; RAS, rat sarcoma; RAF, v-raf murine sarcoma viral oncogene; MEK, mitogen-activated protein kinase kinase; ERK, extracellular signal-regulated kinase.

*BRAF* is involved in mitogen-activated protein kinase (MAPK) pathway that includes the rat sarcoma (RAS)–rapidly accelerated fibrosarcoma (RAF)–mitogen-activated protein/extracellular signal-regulated kinase kinase (MEK)–extracellular signal-regulated kinase (ERK) mitogen-activated protein kinase. After activation of epithelial growth factor receptor (EGFR), RAS–RAF–MEK–ERK pathway will be activated and modulate cell proliferation and survival (11) (**Figure 1**). In normal tissue, the *BRAF* kinase is generally silenced *via* negative feedback once the signal has moved on to the next point in the cascade. However, when *BRAF* mutations occur, the activation of the RAS–RAF–MEK–ERK pathway will be sustained and will lead to uncontrolled cell growth and proliferation; this makes *BRAF* mutations potential oncogenic drivers (12, 13). Generally, *BRAF* mutations commonly present in human cancers with an 8% incidence in all human cancers, predominantly in hairy cell leukemia (100%) (14), melanoma tumors (40%–50%) (15–17), thyroid carcinoma (10%–70%, based on the histologic classification) (18, 19), colorectal cancer (10%) (20, 21), and rarely in lung cancer (1%–2%) (11, 22).

*BRAF* mutations could be divided into three classes based on mutation site. Class I mutants including V600E/K/D/R, which occurs in the valine residue at amino acid position 600 of exon 15, promote constitutive activation of MAPK pathway, causing strong activation of *BRAF* kinase; in addition, this type of mutations often presents high sensitivity to *BRAF* and MEK inhibitors (23, 24). Class II mutants, including K601, L597, G464, and G469 mutations, are located in the activation segment or P-loop and signal as RAS-independent dimers (24, 25).

**Figure 2:**
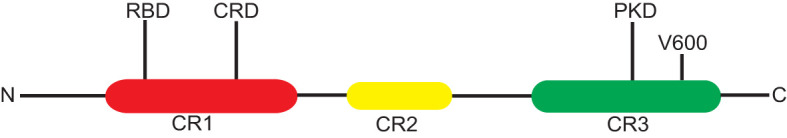
The structure of BRAF gene. N, C, amino and carboxyl end; RBD, Ras-binding domain; CR, conserved region; CRD, cysteine-rich domain; PKD, protein kinase domain; CR1/2/3; conserved region-1/2/3, CR1 contains RBD and CRD, V600E mutation occurs in CR3.

Class III mutants that occur in the P-loop, catalytic loop, or DFG motif have impaired BRAF kinase activity; however, the activity of MAPK pathway signaling is enhanced *via* Raf-1 proto-oncogene CRAF activation (**Figure 2**) (24). All the class II and III mutations are non-V600 mutations, and *BRAF* mutations are usually classified as V600 mutations and non-V600 mutations in routine clinical practice. Actually, approximately 50% of *BRAF* mutations in NSCLC are non-V600 mutations (26–28). In addition, class II and III BRAF mutations are sensitive to current BRAF inhibitors; hence, novel-generation BRAF inhibitors warrant being developed.

## BRAF Deletion Mutations

Several previous studies have demonstrated that *BRAF* deletion mutations can occur in melanoma, pancreatic cancer, and thyroid cancer; in addition, activating *BRAF* deletion mutations might serve as a type of resistance mechanism to *BRAF* inhibitors plus MEK inhibitors (29–31). Generally, deletion mutations happen adjacent to the αC helix in the kinase domain of *BRAF*, resulting in enhanced kinase activity by suppressing the αC helix in its active conformation (29). This type of *BRAF* mutation is similar to class I mutants functioning as RAS-independent monomers (31).

## BRAF Fusions

At least 18 different 5´ fusion partners have been found across different cancer types including NSCLC, and the most common fusion partner is AGK in NSCLC (13, 32). The occurrence rate of *BRAF* fusions is smaller than 1% in NSCLC, and all NSCLCs with *BRAF* fusions were adenocarcinomas or NSCLC with adenocarcinoma features. Most *BRAF* fusion patterns are in-frame with breakpoints on the *BRAF* kinase domain (13, 32). In addition, remarkably, conserved fusions have been reported to occur in 85% of astrocytic pilocytomas (33). Activating *BRAF* fusions occur in truncation of the N-terminal CR1 auto-inhibitory domain, leading to the constitutive activation of *BRAF* pathway that resembles class II BRAF mutants (34). Up to now, limited data have revealed the activities of *BRAF* inhibitors and MEK inhibitors in treating *BRAF* fusion mutations.

## Detection of BRAF Mutations

Single-gene assays for *BRAF* mutations are extensively used across other cancer types including melanoma. The most commonly used assay is RT-PCR. So far, the cobas 4800 *BRAF* V600 Mutation Test and THxID-*BRAF* kit are Food and Drug Administration (FDA)-approved companion diagnostic tests (35–37). In addition, laboratory-developed tests also could be applied to test a patient’s *BRAF* mutation status, although confirmatory tests *via* other methods are necessary. The major advantages of RT-PCR are faster turnaround time, better reproducibility, higher specificity and sensitivity, and lower cost compared with multiple gene sequencing methods. However, most of these methods are merely for *BRAF* V600E mutation located in exon 15. They lack the ability to detect exon 11 mutations that also are seen in NSCLC (38). Hence, next-generation sequencing (NGS) including a multiple gene panel should be applied to evaluate V600E mutation and non-V600E mutations that could happen in exon 11 and exon 15 (15, 26).

The other kind of single-gene test is immunohistochemistry (IHC) for *BRAF* mutations. However, the only available antibody used in IHC for mutant *BRAF* protein is monoclonal antibody VE1. The advantage of this method is to identify a qualitative change (i.e., the presence or absence of the protein), but the accuracy is limited in quantitating changes in expression than other antibody-based assays, such as the enzyme-linked immunosorbent assay (39). The limitation of this test is similar to RT-PCR that only can test *BRAF*-V600E mutation. In addition, only a few cases of lung cancer have shown that VE1 clone has the potential to stain between 90% and 100% of p.V600E-mutant adenocarcinomas (40). It was previously reported that IHC using VE1 antibody is incapable of testing non-V600E mutation (41). However, another study has demonstrated that 599T insertion mutation in 1/21 cases stained with VE1 is positive for VE1 antibody. Hence, no standard recommendation or consensus was obtained for using *BRAF* p.V600E IHC (VE1) testing in NSCLC; extension validation must be deployed when IHC is used to test *BRAF*-V600E mutation.

## Next-Generation Sequencing

As mentioned above, single-gene tests for *BRAF* mutation are unable to identify mutations occurring in exon 11; hence, a multiple-gene panel including *BRAF* mutations is more practical. In addition, with more novel rare driver genes discovered, there is an increased need for multigene testing compared to single-gene approaches. Current guidelines for gene testing in NSCLC should include *BRAF*, *mesenchymal epithelial transition factor receptor (MET)*, *rearranged during transfection (RET)*, *Human Epidermal Growth Factor Receptor 2 (HER2)*, *neurotrophic tropomyosin receptor kinase (NTRK)*, and *Kirsten Rat Sarcoma Viral Oncogene Homolog (KRAS)* for cases in which the common oncogenic drivers (*EGFR*, *anaplastic lymphoma kinase (ALK)*, and *ROS proto-oncogene 1 (ROS1)* are negative and whenever an adequate technique is available (42). The advantages of NGS are as follows: 1) fewer tumor tissue; 2) facilitates testing of multiple biomarkers; 3) includes emerging biomarkers for clinical trial enrollment. Generally, it is more economical than sequential testing (43, 44). However, because of more data, interpreting the NGS reports becomes complex and its availability in the community or rural region is poor. Besides, the turnaround time of NGS is longer than those of RT-PCR and IHC assay. Hence, multiple-gene RT-PCR kit might be a more reasonable choice for gene tests.

## Current Treatment Landscape

### Chemotherapy

The activities of chemotherapy have been fully explored in patients with *BRAF* V600E mutation advanced NSCLC. Documented studies have revealed that advanced NSCLC patients harboring *BRAF* V600E mutations present poor prognosis when administered with chemotherapy; in addition, patients with *BRAF* V600E mutations appear to be insensitive to platinum-based chemotherapy (45–47). However, several reports showed that NSCLC patients harboring *BRAF* V600E mutations seemed to have extended survival compared with patients without oncogenic drivers (47, 48). Additionally, Claire Tissot et al. (49) have reported that patients’ survival is not connected with BRAF mutation status. Another French study also observed similar results that *BRAF* mutation was not prognostic of overall survival (50). In addition, a recent study suggested that class I *BRAF* V600E mutations have the potential to be less aggressive than class II and III non-V600E mutations, which present more possibilities to occur in brain metastases and RAS co-alterations; hence, this specific behavior made non-V600E patients have shorter progression free survival (PFS) and overall survival (OS) to chemotherapy, although the difference might be driven by fewer extrathoracic metastases and higher use of targeted therapies in class I patients (51). However, because of limited cases included in these studies, the results presented here should be interpreted with caution. Hence, future larger randomized trials are urgently warranted.

### Immune Checkpoint Inhibitor Monotherapy

Previous retrospective small-sample studies have found that *BRAF*-mutated NSCLC patients tend to display positive programmed cell death ligand 1 (PD-L1) expression (52–56); however, because of limited cases, no clear correlation between PD-L1 and BRAF mutations were found. Recently, a study including 29 NSCLC patients harboring *BRAF* mutations showed us that approximately 69% (20/29) of patients were PD-L1 positive; among them, over 40% (13/29) of patients presented higher PD-L1 expression (PD-L1 ≥50%). In addition, *BRAF*-mutated NSCLC patients were correlated with low/intermediate tumor mutation burden (TMB) and microsatellite-stable status (57). In this study, researchers have reported that patients harboring *BRAF* mutations displayed limited response to ICIs. Additionally, several retrospective studies also observed a similar phenomenon. The objective response rate (ORR) to single anti–PD-(L)1 agent in BRAF-mutant patients is about 10%–30%, with a median PFS of 2–4 months, which is equal to that of a second-line ICI monotherapy in wild-type NSCLC (57–61) (**Table 1**). Combining these data, we can conclude that ORR and PFS of patients with BRAF non-V600E are higher than those in patients harboring BRAF V600E mutations, but OS results seem paradoxical, potential exploration might be that BRAF V600E mutations could benefit from targeted therapy. On the other hand, non-V600E mutations usually happen in smokers, and smoking status was found to be related to response to immunotherapy (62). In summary, these data indicated limited efficacy of ICIs in BRAF-mutant NSCLC. Recently, a case with BRAF V600E mutation presented durable response to ICI combined chemotherapy with PFS of 20 months (63). This is the first evidence of patients with BRAF V600E alteration treated with ICI combination regimens. This case provided evidence that the ICI combined regimens might be a promising choice for BRAF V600E-mutated NSCLC. Further prospective clinical trials are eagerly needed.

**Table 1:** ICI monotherapy for BRAF-mutated NSCLC.

Trial	Mutation type	Numbers	objective response rate (ORR)	progression free survival (PFS)	overall survival (OS)
**Immunotarget**	V600E	17	NA	1.8	8.2
	Non-V600E	18	NA	4.1	17.2
**Memorial Sloan Kettering Cancer center (MSKCC)**	V600E	10	10	1.4	26
	Non-V600E	36	22	3.2	24
**Isarel lung cancer group (ICLG)**	V600E	12	25	3.7	NA
	Non-V600E	10	33	4.1	
**Expanded Access Program (EAP) Nivolumab**	BRAF	11	9	NA	10.3
**French Lung Cancer Group (GFPC) 01-2018**	V600E	26	26	5.3	22.5
	Non-V600E	18	35	4.9	12

### Targeted Therapy

Sorafenib, an early-generation BRAF inhibitor, was developed as a targeted therapy against BRAF mutant kinase. Sorafenib is an oral multikinase inhibitor that displays activities to target B/C-RAF, Vascular Endothelial Growth Factor Receptor (VEGFR2/3), platelet-derived growth factor receptor (PDGFR-β), and c-Kit (64, 65). Preclinical models suggested that sorafenib could suppress various cancer cell proliferation and tumor growth *via* inhibiting MEK and ERK phosphorylation (64). These studies provide a theoretical basis for sorafenib as a BRAF inhibitor. However, a previous study showed that the antitumor activities of sorafenib are correlated with *EGFR* mutation status but not *K-ras* mutation status (67). Carter et al. (68) have demonstrated that concurrent administration of sorafenib with chemotherapeutics could effectively delay tumor growth without increasing toxicity. These data promoted some researchers who have designed clinical trials to testify the value of sorafenib in NSCLC; however, these trials have not tested the patients’ *BRAF* mutation status (69, 70). Hence, whether sorafenib could serve as a *BRAF* inhibitor remains to be explored.

Dabrafenib and vemurafenib, novel-generation *BRAF* inhibitors, are ATP-competitive inhibitors of *BRAF* kinase. Both agents are specific in targeting *BRAF* V600E mutations. Vemurafenib was initially tested in a “basket” study including multiple non-melanoma cancers with *BRAF* V600 mutants. In the NSCLC cohort, 20 pretreated NSCLC patients were included and achieved a 42% ORR and 7.3 months of PFS (**Table 2**) (71). Gautschi et al. (72) also found that vemurafenib showed promising antitumor activities in *BRAF* V600-mutated NSCLC patients. Additionally, a recent research revealed that vemurafenib was specifically targeting *BRAF*-V600 mutants but was ineffective in patients with *BRAF* non-V600 mutants (73). Combining these data, current lung cancer guidelines recommended that vemurafenib could serve as an optional regimen in certain circumstances. A prospective trial showed that dabrafenib had clinical activity in *BRAF* V600-mutant NSCLC, and dabrafenib might act as a promising treatment choice for patients harboring *BRAF* V600E-mutant NSCLC, which lacks effective treatment options (74). In addition, a recent study has reported that BGB-283, a novel inhibitor of key RAF family kinases, showed promising antitumor activity with acceptable toxicity in patients with *BRAF* V600-mutated solid tumors including NSCLC (75). However, the activity of single *BRAF* inhibitors is limited; hence, researchers began to explore combination therapy. Several studies are ongoing to investigate the novel *BRAF* inhibitors in *BRAF*-mutated NSCLC patients.

**Table 2:** Targeted therapy for BRAF-mutated NSCLC.

Trial	Treatment lines	Agents	ORR	PFS	OS
**NCT01524978**	≥2	Vemurafenib	42%	7.3	NA
**EURAF Cohort**	≥2	Vemurafenib, dabrafenib, or sorafenib	53%	5.0	10.8
**AcSé**	≥2	Vemurafenib	44.9%	5.2	10
**NCT01336634**	≥2	Dabrafenib	33%	5.5	NA
**NCT02610361**	≥2	BGB-283	20%	NA	NA
**NCT01336634**	≥2	Dabrafenib+Trametinib	63%	10.2	18.2
**NCT01336634**	1	Dabrafenib+Trametinib	64%	10.9	24.6
**NCT02974725**	≥2	LXH254+LTT462	66.7%	NA	NA

Dabrafenib plus trametinib, a type of MEK inhibitor, was the first explored combination regimen focusing on BRAF pathway inhibition. A previous phase 2, multicohort, multicenter, non-randomized, open-label study included 36 patients harboring *BRAF* V600E mutant who were treated with first-line dabrafenib plus trametinib. The ORR was 64% and PFS was 14.6 months, as assessed by an independent review committee; in addition, an OS of 24.6 months was achieved (76, 77). This study indicated that dual blockade of the BRAF pathway with BRAF inhibitors and MEK inhibitors could produce a much stronger efficacy. Besides, dabrafenib plus trametinib combination as second-line or later setting was also evaluated (76, 77). Surprisingly, dual blockade of the BRAF pathway achieved similar results compared to that in first-line setting, with 63.2% ORR and almost 10 months of PFS. This study further confirmed the survival advantage of dabrafenib plus trametinib combination compared to single agents. Furthermore, LXH254, a novel BRAF/CRAF inhibitor, plus LTT462, an ERK1/2 inhibitor, was explored to evaluate its activity in patients with advanced/metastatic *K-ras-* or *BRAF*-mutant NSCLC in a phase Ib dose escalation study; preliminary analysis showed signs of efficacy in patients with *BRAF*-mutant NSCLC (78). Dose expansion is ongoing, and further efficacy analysis remains to be seen.

### Immune Checkpoint Inhibitor Combined Therapy

ICIs have transformed the treatment pattern of advanced NSCLC without oncogenic driver mutations. However, the activity of ICIs in NSCLC with oncogenic driver mutations remains limited. Recently, Lu et al. reported a case diagnosed with stage IV NSCLC with *BRAF* V600E mutation that achieved a longer response after being treated with atezolizumab plus chemotherapy (63). This study suggested that ICI combined therapy might be a promising regimen for NSCLC with *BRAF* V600E mutations. In addition, preclinical data revealed that selumetinib and trametinib could improve T-cell activation and increase CTLA-4 expression. Besides, anti-Cytotoxic T lymphocyte associate protein-4 (CTLA-4) antibody plus selumetinib and trametinib presented a survival benefit in mice bearing tumors with *K-ras* mutation (79, 80). Based on the preclinical data, Hellmann et al. (81) have designed a study to investigate the safety and clinical activity of combining a MEK inhibitor, cobimetinib, and a PD-L1 inhibitor, atezolizumab, in patients with solid tumors (n = 152). Among them, 28 NSCLC patients were recruited. For NSCLC patients, the median OS was 13.2 months, and the ORR was 18% (81). Additionally, another phase I/II trial was designed to evaluate the safety and efficacy of durvalumab plus tremelimumab with continuous or intermittent administration of selumetinib in advanced NSCLC patients (82) (**Table 3**). Up to now, clinical trials in melanoma have demonstrated the activities of ICI plus BRAF-targeted therapy; notably, the safety profile of this combination regimen warranted more attention. In addition, for NSCLC, data about ICI-combined *BRAF*-targeted therapies remained limited. The safety and clinical efficacy of this pattern warrant further investigation.

**Table 3:** Several ongoing trials of ICIs combined with targeted therapy.

Trial	Phase	Treatment lines	Experimental arm	Enrolled population	Status
**NCT03600701**	II	≥2	Atezolizumab+combimetinib	Metastatic, Recurrent, or Refractory non-small cell lung cancer	Recruiting
**NCT03299088**	Ib	≥2	Pembrolizumab+trametinib	Stage IV non-small cell lung cancer with *K-ras* gene mutations	Active
**NCT03225664**	Ib/II	≥2	Pembrolizumab+trametinib	Recurrent non-small cell lung cancer	Active

### Mechanisms of Resistance to BRAF Tyrosine Kinase Inhibitors

Exactly as other targeted therapies in NSCLC, resistance to BRAF pathway inhibitors would inevitably occur, leading to disease progression. However, information about resistance mechanisms of BRAF pathway inhibitors is poorly defined.

**Figure 3:**
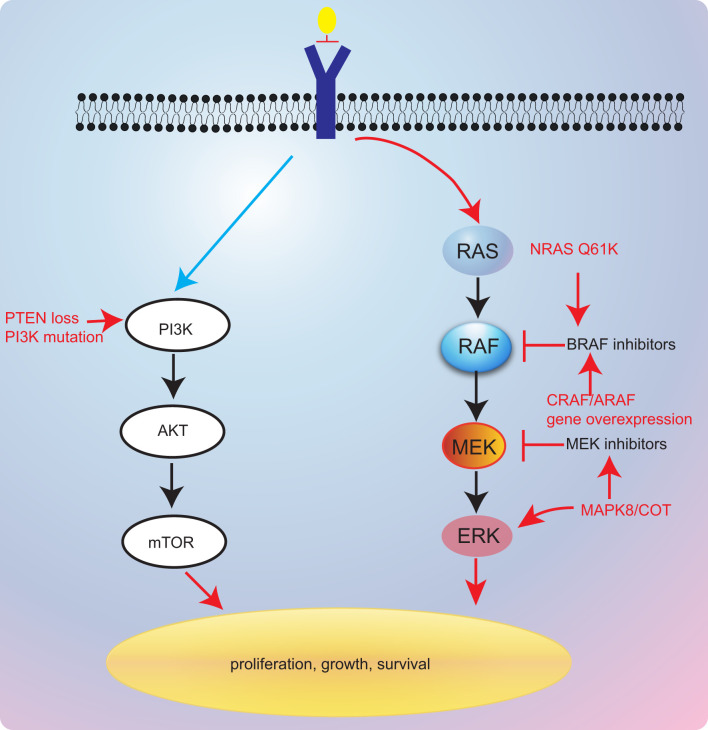
Resistance mechanisms of targeted therapies.

Currently, bypass activation is the main cause of secondary resistance of targeted therapy. However, there is limited report thus far that has revealed the resistance mechanisms of BRAF inhibitors in *BRAF* V600E NSCLC. In melanoma, other isoforms of RAF proteins (CRAF and A-Raf proto-oncogene (ARAF)) could also activate the MAPK pathway when *BRAF* was inhibited, which leads to resistance to BRAF pathway inhibitors (83). Several studies have also demonstrated that MAPK pathway stimulation by MAP3K8 or COT is associated with BRAF inhibitor resistance (83, 84) (**Figure 3**). However, the combination of BRAF inhibitors and MEK inhibitors could effectively reverse the resistance to monotherapy in *BRAF*-mutant NSCLC.

Additionally, Rudin et al. (85) have reported that acquired *K-ras* G12D mutation might be contributing to secondary resistance to dabrafenib. Coincidentally, *K-ras* G12V was also considered as mediating resistance to BRAF inhibitors (86). Besides, in a previous case report, researchers presented a case that was treated with dabrafenib and trametinib that developed *N-ras* Q61K mutation (87). These reports revealed that *RAS* gene might a critical gene modulator in resistance mechanisms to BRAF/MEK inhibitors. The last European Society For Medical Oncology (ESMO) congress reported a novel combination of LXH254 and LTT462 that might overcome RAS-related resistance to BRAF/MEK inhibitors (78). This regimen has shown antitumor activity in *BRAF*-mutant and *K-ras*-mutated patients. However, further investigation remains warranted.

Inactivation of phosphatase and tensin homolog (PTEN), a tumor suppressor, was also found to be involved in resistance to BRAF inhibitors in melanoma (88–90). A previous study has suggested that shorter PFS to anti-BRAF drugs was found in *PTEN*-deficient patients, further supporting the role of *PTEN* in resistance to BRAF inhibitors (91). Notably, *PTEN* lack-of-function alterations may be resistant to dabrafenib–trametinib combinations, the current standard of care, which lacks effective resolutions to this resistance.

## Conclusions

Targeted therapy in driver gene-positive NSCLC has obtained significant progress and greatly revolutionized the landscape of NSCLC. However, the current treatment choice for *BRAF*-mutated NSCLC patients is not satisfactory because of lower incidence. Current guidelines recommend dabrafenib plus tremetinib as the only one standard targeted therapy option for BRAF-mutated NSCLC. However, the underlying resistance mechanisms of this combination regimen have not been clearly defined; in addition, current targeted therapy specifically targeted to *BRAF* V600E mutation exhibited poorer efficacy against non-V600E mutation.

Furthermore, clinical investigations will be also confronted with ongoing challenges. Firstly, randomized prospective phase III trials are difficult to conduct owing to the low incidence of *BRAF* mutant-positive NSCLC. Secondly, the utility and ethics of randomizing patients to a control arm with poorer efficacy and shorter survival durations are controversial. In addition, several studies have demonstrated that ICIs could show efficacy in this population; the problem that lies ahead is which regimen should be given first.

In the future, the activity of chemoimmunotherapy and combinations of TKIs with chemotherapy, anti VEGF/VEGFR agents, and/or immunotherapy in patients with *BRAF*-mutated cancers needs to be determined. In addition, the development of agents targeting non-V600E mutations should speed up.

## Author Contributions

(I) Conception and design: Ningning Yan, Shujing Shen, and Xingya Li. (II) Article writing: All authors. (III) Final approval of article: All authors.

## Funding

This work was funded by the joint construction project of Henan Province and Ministry (No. LHGJ20190013).

## Conflict of Interest

The authors declare that the research was conducted in the absence of any commercial or financial relationships that could be construed as a potential conflict of interest.

## Publisher’s Note

All claims expressed in this article are solely those of the authors and do not necessarily represent those of their affiliated organizations, or those of the publisher, the editors and the reviewers. Any product that may be evaluated in this article, or claim that may be made by its manufacturer, is not guaranteed or endorsed by the publisher.
